# 702 Outcomes of COPD Patients with Burn Injuries at a Single Institution

**DOI:** 10.1093/jbcr/irac012.256

**Published:** 2022-03-23

**Authors:** Sanja Sljivic, Lori Chrisco, Rabia Nizamani, Booker King, R O B E R T W Matthews, Felicia Williams

**Affiliations:** University of North Carolina - Chapel Hill, Chapel Hill, North Carolina; North Carolina Jaycee Burn Center, Chapel Hill, North Carolina; UNC Jaycee Burn Center, Chapel Hill, North Carolina; UNC Jaycee Burn Center, Chapel Hill, North Carolina; UNC, Apex, North Carolina; UNC Jaycee Burn Center, Chapel Hill, North Carolina

## Abstract

**Introduction:**

Chronic obstructive pulmonary disease (COPD) is a condition with significant morbidity and mortality. In 2018, about 16 million adults in the United States reported a diagnosis of COPD based on data from the American Lung Association. Home oxygen is often used in more severe cases of COPD, and despite warnings against smoking while using home oxygen, many patients continue to sustain burn injuries. An existing diagnosis of COPD can further complicate management of a burn patient, especially if there is concomitant inhalation injury present. The objective of this study was to explore the outcomes of COPD patients admitted to our Burn Center.

**Methods:**

This was a single-site, retrospective review using our institutional Burn Center registry. All adult patients with flame burns (18 years or older) admitted to our Burn Center between July 1, 2011 and June 30, 2020 who had a history of COPD with and without home oxygen use were included in this study. All adult patients with flame burns, who did not have a history of COPD, were included for comparative purposes. Variables of interest included demographics, burn mechanism, length of stay (LOS), ICU and ventilator days, and mortality.

**Results:**

There were a total of 4,397 patients with flame burns included in this study, and 515 of those patients were identified to have an existing diagnosis of COPD. The mean age of the COPD group was 45.1 years +/- 13.0 years, and the patient population was predominantly male (60.4%). The mean total body surface area (TBSA) involvement was 5.12% +/- 10.38%. Inhalation injury was present in 10.1% of patients with COPD and in 7.8% of those without COPD. The mean LOS for the COPD group was 11.9 days +/- 19.4 days and 13.4 days +/- 31.0 days for the non-COPD group. The mean ICU LOS for the COPD group was 11.2 days +/- 19.9 days and 18.0 days +/- 37.0 days for the non-COPD group. The mean number of ventilator days was 16.5 days +/- 35.4 days for the COPD group and 26.3 days +/- 42.0 days for the non-COPD group. The overall hospital mortality was 10.3% for the COPD group and 4.3% for the non-COPD group.

**Conclusions:**

This study demonstrates that the overall hospital mortality was highest in the COPD group. Although hospital and ICU length of stay, as well as the number of ventilator days were higher in the non-COPD group, it remains clear that an existing diagnosis of COPD can negatively impact the outcomes of burn patients.

